# Randomized Controlled Trial of the Cholera-Hospital-Based-Intervention-for-7-Days (CHoBI7) Cholera Rapid Response Program to Reduce Diarrheal Diseases in Bangladesh

**DOI:** 10.3390/ijerph191912905

**Published:** 2022-10-08

**Authors:** Christine Marie George, Tahmina Parvin, Md. Sazzadul Islam Bhuyian, Ismat Minhaj Uddin, Fatema Zohura, Jahed Masud, Shirajum Monira, David A. Sack, Jamie Perin, Munirul Alam, A. S. G. Faruque

**Affiliations:** 1 Department of International Health, Johns Hopkins Bloomberg School of Public Health, Baltimore, MD 21205-2103, USA; 2 Research Training and Management International, Dhaka 1216, Bangladesh; 3 International Centre for Diarrhoeal Disease Research, Bangladesh (icddr,b), Dhaka 1212, Bangladesh

**Keywords:** randomized controlled trial, Bangladesh, cholera, water, sanitation, hygiene, program evaluation, diarrheal diseases, community members

## Abstract

(a) Objective: To build an evidence base on effective water, sanitation, and hygiene interventions to reduce diarrheal diseases in cholera hotspots, we developed the CHoBI7 Cholera Rapid Response Program. (b) Methods: Once a cholera patient (confirmed by bacterial culture) is identified at a health facility, a health promoter delivers a targeted WASH intervention to the cholera hotspot (households within 20 m of a cholera patient) through both in-person visits during the first week and bi-weekly WASH mobile messages for the 3-month program period. A randomized controlled trial of the CHoBI7 Cholera Rapid Response Program was conducted with 284 participants in 15 cholera hotspots around cholera patients in urban Dhaka, Bangladesh. This program was compared to the standard message in Bangladesh on the use of oral rehydration solution for dehydration. Five-hour structured observation of handwashing with soap and diarrhea surveillance was conducted monthly. (c) Findings: Handwashing with soap at food- and stool-related events was significantly higher in the CHoBI7 Cholera Rapid Response Program arm compared to the standard message arm at all timepoints (overall 54% in the CHoBI7 arm vs. 23% in the standard arm, *p* < 0.05). Furthermore, there was a significant reduction in diarrheal prevalence for all participants (adults and children) (Prevalence Ratio (PR) 0.35, 95% CI: 0.14–0.85) and for children under 5 years of age (PR: 0.27, 95% CI: 0.085–0.87) during the 3-month program. (d) Conclusions: These findings demonstrate that the CHoBI7 Cholera Rapid Response Program is effective in lowering diarrhea prevalence and increasing handwashing with soap for a population at high risk of cholera.

## 1. Introduction

Globally, 2.9 million cholera cases and 95,000 cholera deaths are estimated to occur annually in cholera-endemic countries. In Bangladesh alone, there are estimated to be over 160,000 cholera cases and 5000 deaths each year [1]. Beyond cases, an additional 84.5 million people in Bangladesh live in districts classified as high risk for cholera (annual incidence rate > 3/1000) [2]. Bangladesh has bimodal peaks for cholera. One peak in the spring (March–April) and one in the fall (October–November); however, in areas such as the capital city of Dhaka, cholera cases occur year-round [3,4]. Previous studies have found unimproved water sources, open containers for drinking water storage, consumption of food outside of the home, lack of water treatment, unimproved sanitation, and lack of handwashing with soap to be risk factors for symptomatic cholera infections [5,6,7,8].

Individuals living in close proximity to cholera patients are at an increased risk of subsequent cholera infections [9,10,11]. The period of highest risk is the first week after the cholera patient presents at a health facility, with elevated cholera risk continuing up to one month [9,10,11,12]. Our previous study in Bangladesh found that individuals living within 50 m of a cholera patient were at >30 times higher risk of developing a cholera infection that required hospitalization during this one-week window compared to the general population [10]. Despite this growing evidence, there has been little work done to develop and evaluate targeted interventions to reduce cholera among this high-risk population living in cholera hotspots.

The standard of care in Bangladesh for cholera patients at the time of discharge from the health facility is to give these patients oral rehydration solution (ORS) for rehydration. There is no standard of care for the family members of cholera patients despite them being at >100 times higher risk of developing cholera infections than the general population during the one-week period after the index patient seeks care at a health facility [11,12,13]. Therefore, in an effort to develop a standard of care for household members of cholera patients in Bangladesh, our research group developed the Cholera Hospital-Based Intervention for 7 days (CHoBI7). This targeted water, sanitation, and hygiene (WASH) intervention focuses on promoting handwashing with soap and water treatment to cholera patients and their household members during the 7-day period they are at the highest risk for cholera infections after the cholera patient presents at a health facility.

To evaluate the efficacy of the targeted CHoBI7 WASH intervention in reducing cholera infections among household members of cholera patients during their 7-day high-risk period, we conducted a randomized controlled trial (RCT) of CHoBI7 from 2013 to 2014. We compared the CHoBI7 WASH intervention to the standard recommendation given in Bangladesh to diarrhea patients at discharge on ORS use. Delivery of the CHoBI7 intervention to cholera patient households resulted in a 47% reduction in overall cholera infections and a significant reduction in symptomatic cholera during the 7-day high-risk period for subsequent cholera infections [14]. Our findings were consistent with a recent study in the Democratic Republic of the Congo (DRC), which found that suspected cholera patients provided with a hygiene kit containing water treatment tablets and soap had a significantly lowered incidence of suspected cholera [15].

To expand the evidence base on CHoBI7 further to those residing in cholera hotspots around cholera patients, in the present study, we developed the CHoBI7 Cholera Rapid Response Program. For this program, when a cholera patient confirmed by bacterial culture is identified at a health facility, a health promoter delivers a targeted WASH intervention to the cholera hotspot where the patient resides (areas within 20 m of cholera patient households) through both in-person visits during the 7-day high-risk period and bi-weekly WASH mobile messages for the 3-month program period. In this study, we conducted an RCT of the CHoBI7 Cholera Rapid Response Program to evaluate the efficacy of this intervention in reducing diarrheal diseases and increasing handwashing with soap and water treatment behaviors.

## 2. Methods

### 2.1. Study Design

The CHoBI7 Cholera Rapid Response Program RCT was a two-arm cluster RCT conducted in urban Dhaka, Bangladesh, from 22 March 2021 to 28 November 2021, where a cholera hotspot was a ring of 20 m around the residence of a cholera patient. All eligible households meeting our study eligibility criteria were enrolled. A 20-m ring was selected to include households at the highest risk for subsequent cholera infections [9]. Cholera patients were identified through the International Centre for Diarrhoeal Disease Research, Bangladesh (icddr,b) diarrheal surveillance system, where a stool sample was collected from every 50th diarrheal patient admitted to the health facility and analyzed for a panel of enteric pathogens by bacterial culture and microscopy. *Vibrio cholerae* was one of the enteric pathogens assessed by bacterial culture through this diarrheal surveillance system. The cluster RCT compared the standard recommendation given in Bangladesh to diarrhea patients at discharge from health facility staff on ORS use for rehydration (standard message arm) to the CHoBI7 Cholera Rapid Response Program. The trial is registered at ClinicalTrials.gov (NCT04816552).

### 2.2. Patient and Public Involvement

This study did not include the public in the design, or conduct, reporting, or dissemination plans. Patients and those living in cholera hotspots contributed to intervention design through in-depth interviews conducted during formative research reported elsewhere (Zohura et al. 2022 submitted).

### 2.3. Ethical Approval

All study procedures were approved by the research Ethical Review Committee of International Centre for Diarrhoeal Disease Research, Bangladesh (icddr,b), Dhaka, and the Institutional Review Board of The Johns Hopkins Bloomberg School of Public Health (Baltimore, MD, USA). All study participants provided informed consent. Adult participants (>18 years of age) signed an informed consent and/or parental consent form, and children 12–17 years of age signed an assent form. If a study participant could not read, the consent form was read to him or her, and the participant was then asked to document his or her consent with an X in the presence of a witness. All evaluation and intervention team members received intensive training when the study began and then quarterly refresher trainings to reduce the likelihood of bias in intervention and evaluation activity delivery.

### 2.4. Participants 

In order to be eligible for the trial, households had to: (1) not have running water inside their home (mostly slum areas of Dhaka); (2) report ownership of an active mobile phone in their possession on the day of enrollment; and (3) have a child under five years of age in their household (to assess the impact of the intervention on pediatric diarrheal disease prevalence). 

### 2.5. Randomization and Masking

The study biostatistician (JP) assigned randomization using the software R version 3.3.0. JP did not have involvement in data collection activities for the trial. Block randomization of cholera hotspots was performed based on a randomly permuted block of size 2 prior to the start of the RCT using a random number generator. The study arm assignment was revealed to the study coordinator by the study biostatistician. Blinding was not possible because the intervention had visible components. To minimize bias, we used two separate teams for intervention and evaluation activities. 

### 2.6. Intervention Procedures 

Both the standard recommendation and CHoBI7 Cholera Rapid Response Program arms received the standard recommendation given in Bangladesh to diarrhea patients at discharge from health facility staff on ORS use for rehydration using a pamphlet produced by the government of Bangladesh. The CHoBI7 Cholera Rapid Response Program was developed through a theory-based approach informed by the Integrated Behavioral Model for Water, Sanitation, and Hygiene (IBM-WASH) and the Risks, Attitudes, Norms, Abilities, and Self-regulation (RANAS) Model [16,17]. A detailed description of intervention development is published elsewhere (Zohura et al. 2022 submitted). The CHoBI7 Cholera Rapid Response Program targets the following WASH behaviors: (1) preparing soapy water using water and detergent powder; (2) handwashing with soap at food- and stool-related events; (3) treating household drinking water using chlorine tablets during the 7-day high-risk period after the diarrhea patient was identified in the cholera hotspot; (4) safe drinking water storage in a water vessel with a lid and tap; and (5) heating of household drinking water until it reaches a rolling boil (large bubbles form) after the 7-day high-risk period.

The CHoBI7 Cholera Rapid Response Program has three components for intervention delivery: (1) in-person visits (two home visits and one community session); (2) a cholera prevention package; and (3) weekly mHealth messages for 3 months. The two home visits and one community session (with all enrolled cholera hotspot participants) are delivered by health promoters during the 7-day high-risk period after the cholera patient is identified in a cholera hotspot, within 48–72 h of cholera patient identification at the health facility. In-person intervention visits included the delivery of a flipbook explaining cholera transmission pathways through narratives of stories of actual families and modules explaining the importance of and how to properly wash hands with soap and water, how to treat drinking water using chlorine tablets and boiling, and how to safely store drinking water. The cholera prevention package provided to each household contains the following items: a 7-day supply of chlorine tablets for water treatment, a soapy water bottle containing water and detergent powder (a low-cost alternative to bar soap), a handwashing station, and a water vessel with a lid and tap to ensure safe water storage. WASH mHealth messages, including voice, interactive voice response (IVR), and text messages, are sent bi-weekly for 3 months. These mobile messages are sent by study staff using the web-based VIAMO mobile platform to all phone numbers provided by study households. Two characters deliver the CHoBI7 mHealth messages: Dr. Chobi and Aklima [18]. Dr. Chobi is a doctor at a local hospital who calls and texts participants to share information and reminders on handwashing with soap, water treatment, and safe drinking water storage behaviors. Aklima is a woman who brought her child to a health facility for diarrhea treatment and who learned proper handwashing with soap and water treatment behaviors from Dr. Chobi’s voice, IVR, and text messages. 

### 2.7. Evaluation Procedure

Diarrhea was defined as at least three or more loose stools over a 24-h period in the preceding week. Clinical surveillance for diarrhea was performed at baseline enrollment and monthly thereafter until the 3-month time point for all enrolled household members through in-person visits by evaluation research assistants. For children under 12 years of age, diarrhea was assessed through caregiver reports. In order to observe handwashing behaviors, 5-h structured observation was conducted in households from 7:30 a.m. to 12:30 p.m. at 1 week and 1 and 3 months after enrollment. Handwashing with soap was recorded at food- and stool-related events. To assess the presence of soapy water on the household compound (promoted as part of the intervention activities) and the presence of free available chlorine in household stored drinking water (a measure of household water treatment practices), unannounced spot checks were performed in all households at 1 week and 1 and 3 months after enrollment.

### 2.8. Outcomes

Primary outcomes were: (1) individuals handwashing with soap at food- and stool-related events during 5-h structured observation (a measure of handwashing compliance) (for all individuals in the household 2 years of age or older) at week 1 and 1 and 3 months after enrollment; and (2) presence of chlorine > 0.2 mg/L in household stored drinking water at week 1 and 1 and 3 months after enrollment. (Secondary outcomes were: (1) the prevalence of diarrhea among all age groups and children under 5 years over the 3-month study period based on monthly clinical surveillance; (2) presence of soapy water on the household compound at week 1 and 1 and 3 months after enrollment.

### 2.9. Statistical Analysis

Our original sample size was 300 households from 60 cholera hotspots; however, due to the ongoing COVID-19 pandemic stopping study activities due to lockdowns in 2021, we only achieved recruitment of 73 households from 15 cholera hotspots. This power calculation for the primary outcome is described in Supplementary File S1. We analyzed participants according to their randomized assignment (intention-to-treat). The count of diarrhea episodes as well as the count of those who did not have diarrhea was summarized for each hotspot (to account for the cluster-randomized design) and analyzed with Poisson regression, using an offset for the total number of surveillance visits in each hotspot. This regression model estimated the log diarrhea prevalence ratio for the intervention arm relative to those in the standard message arm, along with an approximate standard error and 95% confidence interval. Handwashing with soap, water treatment, and the presence of soapy water was similarly summarized at the hotspot level, and logistic regression was used to estimate the odds of handwashing with soap and the relative odds of handwashing with soap for the intervention arm compared to the standard message arm. Analyses were performed in R (version 4.2, R Team, Vienna, Austria). 

## 3. Results

**Figure 1:**
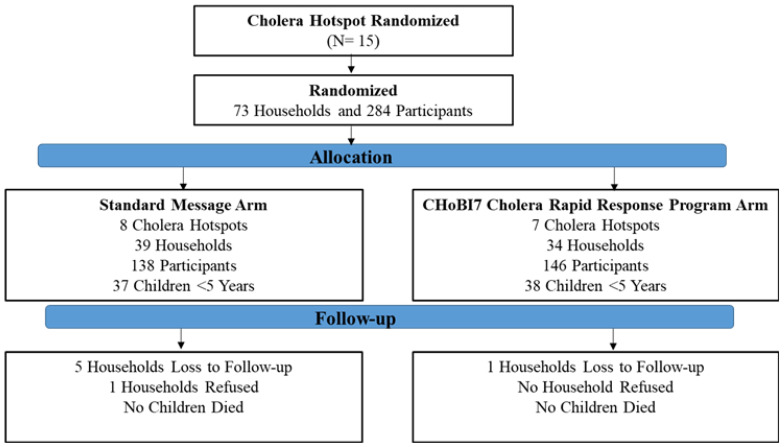
Trial profile and analysis populations for primary outcomes.

Between March and November 2021, we randomly allocated 7 cholera hotspots to the CHoBI7 Cholera Rapid Response Program arm and 8 cholera hotspots to the standard message arm (15 cholera hotspots total) with a total of 284 participants from 73 households (Figure 1). Eight percent of households (n = 6) were lost to follow-up during the 3-month study period. The baseline characteristics of enrolled households were similar across the study arm (Table 1).

**Figure 2:**
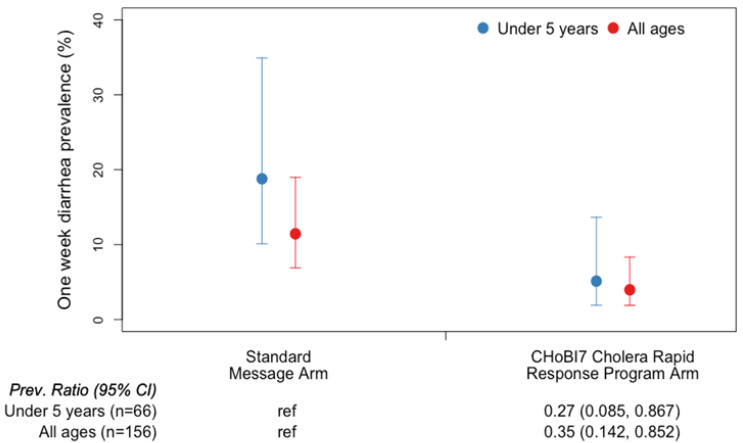
3-Month Diarrhea Prevalence by Study Arm.

Compared to the standard message arm, all age groups combined (children and adults) had significantly lower 3-month diarrhea prevalence in the CHoBI7 Cholera Rapid Response Program arm (prevalence ratio [PR]: 0.35 [95% confidence interval (CI): 0.14–0.85]) (mean diarrhea prevalence: 11% (standard arm) vs. 4% (CHoBI7 arm)). Children <5 years of age also had significantly lower diarrhea prevalence in the CHoBI7 Cholera Rapid Response Program arm compared to the standard message arm (PR: 0.27 [95% CI: 0.09–0.87]) (mean diarrhea prevalence: 19% vs. 5%) (Table 2 and Figure 2). 

Handwashing with soap at food- and stool-related events was significantly higher in the CHoBI7 Cholera Rapid Response Program arm compared to the standard message arm at all timepoints (Table 3) (overall handwashing with soap for all timepoints combined: 54% in the CHoBI7 arm vs. 23% in the standard arm). This higher handwashing with soap was sustained to the 3-month follow-up with 44% of participants in the CHoBI7 Cholera Rapid Response Program arm handwashing with soap at food- and stool-related events compared to 22% in the standard message arm (Odds ratio: 2.78 [95% CI: 1.17, 6.58]). 

Compared to the standard message arm, where no households had a soapy water bottle present on the household compound, the CHoBI7 Cholera Rapid Response Program arm had 97% (33/34) households at Week 1, 92% (24/26) of households at Month 1, and 91% (21/23) of households at Month 3 with a soapy water bottle present during unannounced spot checks on the household compound (*p* < 0.001 for all timepoints). CHoBI7 Cholera Rapid Response Program arm households also had significantly higher free available chlorine >0.2 mg/L in stored drinking water at Week 1 (59% vs. 0%, *p* < 0.001) compared to the standard message arm, however not at Month 1 (35% vs. 0%, *p* = 0.15) (after the 7-day supply of chlorine tablets was completed). Neither study arm had chlorine present > 0.2 mg/L in stored drinking water at the 3-month follow-up.

## 4. Discussion

Delivery of the CHoBI7 Cholera Rapid Response Program in Bangladesh resulted in a significant reduction in diarrhea prevalence and improvements in handwashing with soap and water treatment behaviors over the 3-month program period. This is the first RCT, to our knowledge, of a cholera rapid response program for those living in cholera hotspots in close proximity to cholera patients. These findings built on our previous work, which focused on diarrhea patient households, where we found that CHoBI7 was effective in reducing cholera and diarrhea prevalence for the household members of diarrhea patients [14,19]. Our findings suggest that the CHoBI7 Cholera Rapid Response Program can serve as a promising approach to reducing diarrheal diseases and increasing WASH behaviors in cholera hotspots in slum areas of Dhaka, Bangladesh. 

A key factor contributing to the success of the CHoBI7 Cholera Rapid Response Program was likely the timing of intervention delivery when there was a cholera patient in a nearby household. This was likely a time when perceived susceptibility to cholera was high as well as the perceived benefits of practicing WASH behaviors, resulting in greater WASH behavior change [20]. Previous studies have found increased demand for water treatment products during cholera and severe diarrheal disease outbreaks [21,22]. In Dhaka, Bangladesh, in 2013, utilization of point-of-use chlorine dispensers peaked after cholera-related deaths (Leanne Unicomb, unpublished personal communication). 

The rates of handwashing with soap at food- and stool-related events observed in the CHoBI7 Cholera Rapid Response Program arm in the present study were comparable to those found in our recent RCT of the CHoBI7 mHealth program, which solely focused on diarrhea patient households (Week 1 Follow-up: 71% [previous study] vs. 60% [present study], Month 1: 52% vs. 58%, Month 3: 58% vs. 44%). This finding suggests that there are not large differences in WASH behaviors with the delivery of CHoBI7 between these two different populations at high risk of diarrheal diseases. This was surprising given that one would suspect higher WASH behaviors in households where a family member had severe diarrhea compared to those that have only a neighboring household with severe diarrhea. Future mixed method studies that explore the psychosocial factors driving WASH behaviors among these two populations in parallel are needed to understand further the reason for this finding. 

Rapid delivery of cholera response programs after a cholera patient is identified in an area is crucial to the success of program implementation. A previous study in Haiti found that cholera rapid response teams that were delivered quicker could reduce the number of suspected cholera cases and shorten the outbreak duration [23]. However, previous studies in Nepal and Yemen found that the time required for governmental approval for cholera response efforts after cholera case identification severely delayed the response time for cholera rapid response teams [24]. In the present study, the CHoBI7 Cholera Rapid Response Program was typically delivered in cholera hotspots within 48–72 h of cholera patients being admitted to a health facility, allowing for a rapid response in cholera hotspots. Coordination between government and private health facilities and governing administrative bodies such as Ministries of Health will be important to ensure the timely delivery of cholera rapid response programs.

In the present study, we focused on households within 20 m of cholera patients. There is substantial variability in the cluster size for the delivery of cholera rapid response programs [24]. In DRC, a recent study focused on a grid of, on average, 20–30 households [25]. While in Yemen, Haiti, and Nepal, a ring size of 50 to 100 m was used, and in Zimbabwe, a floor of an apartment building was used in an urban setting [24]. Future studies are needed that evaluate the optimal cluster size for delivery of WASH programs aiming to reduce cholera transmission in hotspots for cholera.

This study has some limitations. First, our sample size was much smaller than planned because of lockdowns in 2021 due to the ongoing COVID-19 pandemic. Nevertheless, the CHoBI7 Cholera Rapid Response Program was found to be effective in reducing diarrhea and WASH behaviors even with this much smaller than expected sample size. Second, we conducted our study in an urban setting which limits the generalizability of our findings to rural settings. Third, we did not measure *Vibrio cholerae* in the stool of participants living in cholera hotspots. We have an ongoing separate randomized controlled trial that will evaluate the impact of the CHoBI7 program on reducing cholera for those living in cholera hotspots nearby cholera patients.

This study has several strengths. First is defining cholera hotspots as locations where a bacterial culture-confirmed cholera patient resided within 20 m, building on previous studies that define cholera hotspots based on suspected cholera cases. Second, our clinical surveillance included both adults and children, building on previous cholera studies that excluded diarrhea for children under 5 years of age. Third is performing structured observation of handwashing with soap and unannounced spot checks of chlorine concentrations and soapy water bottles at multiple timepoints, which allowed us to observe these WASH behaviors prospectively over time.

## 5. Conclusions

The CHoBI7 Cholera Rapid Response Program significantly lowered diarrhea and increased handwashing with soap and water treatment behaviors over our 3-month study period. These promising findings suggest that delivering the CHoBI7 Cholera Rapid Response Program to households in cholera hotspots in urban Bangladesh can serve as an effective approach to increase WASH behaviors and reduce diarrheal diseases among this high-risk population. Future studies are needed that investigate the impact of this program on cholera transmission in this setting. 

## Figures and Tables

**Table 1:** Baseline Population Characteristics by Study Arm.

	Standard Message Arm			CHoBI7 Cholera Rapid Response Program Arm			*p*-Value
	% or N	n	N	% or N	n	N	
**Number of Cholera Hotspots**	8			7			
**Study Households**	39			34			
**All Study Participants**	138			146			
**Baseline Household Member Age (Years)**							
Median ± SD (Min − Max)	21.0 ± 15.3 (0.3–65)			23.7 ± 17.5 (0.2–70)			0.17
0–5 Years	27%	37	138	26%	38	146	
5–17 Years	19%	26	138	14%	20	146	
18 Years or Greater	54%	75	138	60%	88	146	
**Gender**							
Female	52%	72	138	53%	77	146	0.83
**Household Roof Type**							
Tin	72%	28	39	68%	23	34	0.15
Concrete	28%	11	39	32%	11	34	
Other	0%	0	39	0%	0	34	
**Household Wall Type**							
Concrete	97%	38	39	97%	33	34	0.92
Mud	0%	0	39	0%	0	34	
Tin	3%	1	39	3%	1	34	
Other	0%	0	39	0%	0	34	
**Household Floor Type**							
Concrete	100%	39	39	100%	34	34	1.00
Other	0%	0	39	0%	0	34	
**Electricity**	97%	38	39	97%	33	34	0.92
**Refrigerator Ownership**	46%	18	39	62%	21	34	0.18

SD = Standard Deviation.

**Table 2:** 3 Month Diarrhea Prevalence by Study Arm and Age Group.

Study Arm	N	Mean Diarrhea Prevalence †	Prevalence Ratio (95% CI) *
**Standard Message Arm**			
0–5 Years	30	19%	(reference)
All Age Groups	76	11%	(reference)
**CHoBI7 Cholera Rapid Response Program Arm**			
0–5 Years	36	5%	0.27 (0.09–0.87)
All Age Groups	80	4%	0.35 (0.14–0.85)

† Diarrhea (3 or more loose stools) reported in the past week. * Adjusted for follow-up timepoint.

**Table 3:** Individuals Handwashing with Soap at Stool/Toilet or Food Related Events During 5-Hour Structured Observation (Individuals Over 2 Years).

	%	OR (95% CI)	%	OR (95% CI)	%	OR (95% CI)
Individuals Handwashing with Soap at Stool/Toilet or Food Related Events During 5-Hour Structured Observation *						
Standard Message Arm	28%	-	12%	-	22%	-
CHoBI7 Cholera Rapid Response Program Arm	60%	3.94 (2.12, 7.30)	56%	9.57 (3.03, 30.25)	44%	2.78 (1.17, 6.58)
**Presence of Soapy Water in Household**						
Standard Message Arm	0%	-	0%	-	0%	-
CHoBI7 Cholera Rapid Response Program Arm	97%	‡	92%	‡	91%	‡
**Chlorine in Stored Household Drinking Water > 0.2 mg/L**						
Standard Message Arm	0%	-	0%	-	0%	-
CHoBI7 Cholera Rapid Response Program Arm	59%	‡	35%	‡	0%	‡

OR: Odds Ratio; CI: Confidence Interval; ‡: No events in standard message arm OR could not be calculated * Individuals Over 2 Years.

## Data Availability

The data supporting the conclusions of this article are available from the corresponding author upon reasonable request.

## Supplementary Materials

The following supporting information can be downloaded at: https://www.mdpi.com/article/10.3390/ijerph191912905/s1, File S1: Power Calculation and Statistical Analysis.
