# Supplementary material for: Randomized Controlled Trial of the Cholera-Hospital-Based-Intervention-for-7-Days (CHoBI7) Cholera Rapid Response Program to Reduce Diarrheal Diseases in Bangladesh
Source: Int J Environ Res Public Health. 2022 Oct 8;19(19):12905. doi: 10.3390/ijerph191912905 (PMC9566036; doi:10.3390/ijerph191912905)
Supplement: Supplementary file 1 [file ijerph-19-12905-s001.zip › ijerph-1892597-supplementary.pdf]

Supplementary File S1. Power Calculation and Statistical Analysis

Our original sample size of 300 households was based on the largest number of households we could recruit based our budget. However due to COVID this sample size was not met. Our original power calculation is summarized below.
*Assuming an intra-class correlation of 0.1 for handwashing with soap of individuals in the same household, for a design effect of 1.3.

**Power calculation. Detectable differences for outcomes between intervention and comparison arm for 80% power, assuming 10% loss to follow-up among 300 enrolled households resulting in 270 households at the 12 month follow-up**			
**Outcomes**	**N**	**Anticipated %**<br>**in Control Arm**<br>(Based on Current Trial)	**Detectable Difference Between Intervention and Control Arms**
% Individuals handwashing with soap at a stool or food related event during structured observation (100 randomly selected households per timepoint (400 participants at each visit with 10% loss to follow-up))*	360	25%	16%
